# Supplementary material for: Transperitoneal vs extraperitoneal radical cystectomy: A systematic review and meta-analysis
Source: PLoS One. 2023 Nov 30;18(11):e0294809. doi: 10.1371/journal.pone.0294809 (PMC10688672; doi:10.1371/journal.pone.0294809)
Supplement: S1 Fig — (DOCX) [file pone.0294809.s001.docx]

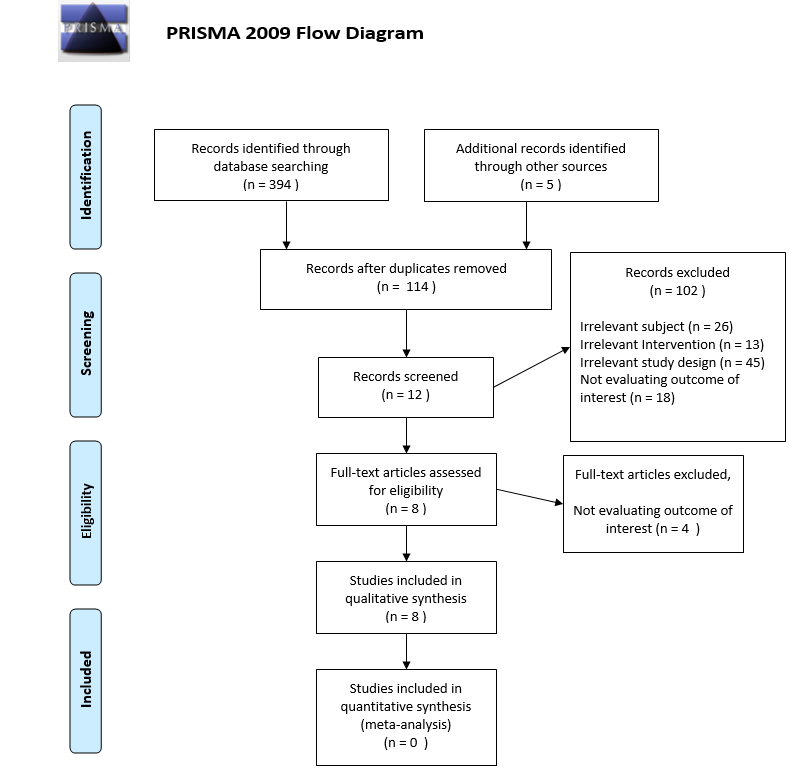


Records identified through databases searching

(n = 250)

Records after duplicates removed

(n = 197)

Records excluded (n = 173)

- Irrelevant subjects and population
- Irrelevant intervention

Records screened

(n = 24)

Records excluded (n = 6)

Articles cannot be fully accessed

Full-text assessed for eligibility

(n = 18)

Records excluded (n = 10)

Not evaluating outcome of interest, combine method of intervention

**S2 Figure. PRISMA flow diagram**

Studies included in this study

(n = 8)
