# Supplementary material for: Transperitoneal vs extraperitoneal radical cystectomy: A systematic review and meta-analysis
Source: PLoS One. 2023 Nov 30;18(11):e0294809. doi: 10.1371/journal.pone.0294809 (PMC10688672; doi:10.1371/journal.pone.0294809)
Supplement: S2 Fig — (DOCX) [file pone.0294809.s002.docx]

**
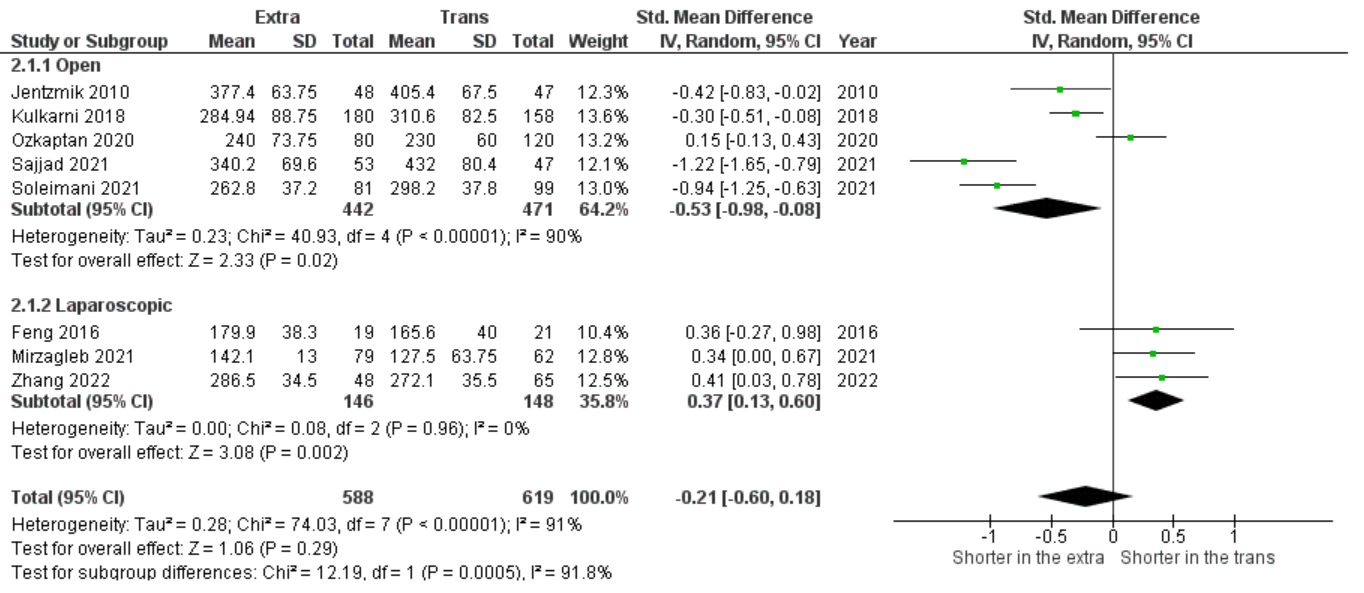
**

**Fig 1.** Operative time (minutes) in extraperitoneal vs transperitoneal approach. CI, confidence interval; IV, inverse variance; SD, standard deviation

**
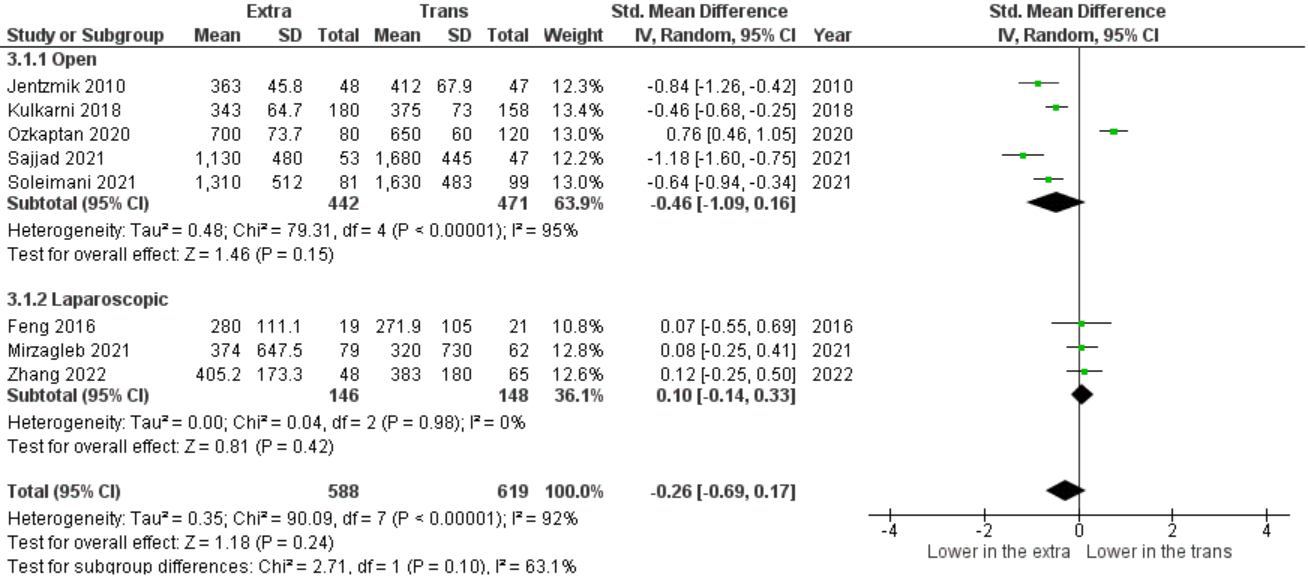
**

**Fig 2.** Estimated blood loss (mL) in extraperitoneal vs transperitoneal approach. CI, confidence interval; IV, inverse variance; SD, standard deviation

**
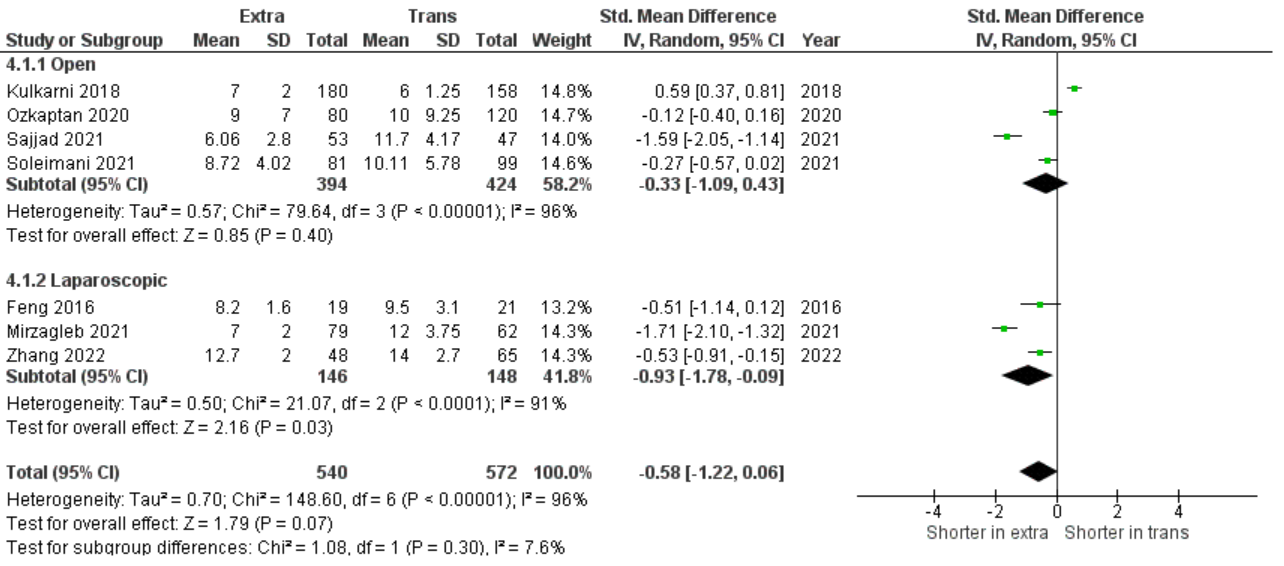
**

**Fig 3.** Hospital stays (days) in extraperitoneal vs transperitoneal approach. CI, confidence interval; IV, inverse variance; SD, standard deviation

**
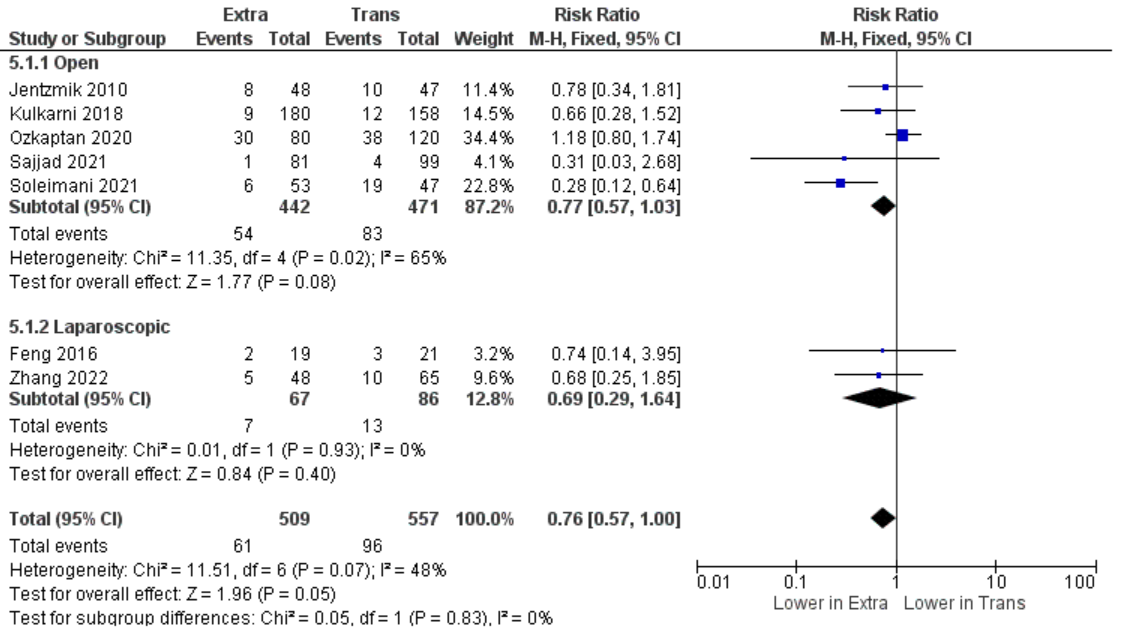
**

**Fig 4.** Total infection (n) in extraperitoneal vs transperitoneal approach. CI, confidence interval; IV, inverse variance; SD, standard deviation

**
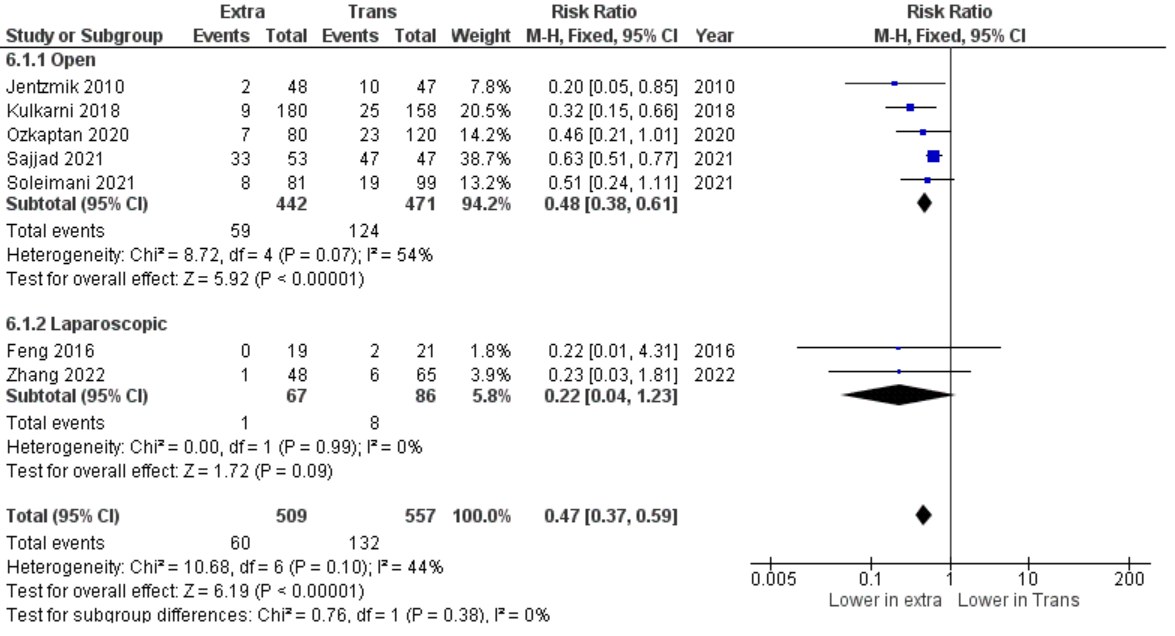
**

**Fig 5.** Post-operative ileus (n) in extraperitoneal vs transperitoneal approach. CI, confidence interval; IV, inverse variance; SD, standard deviation

**
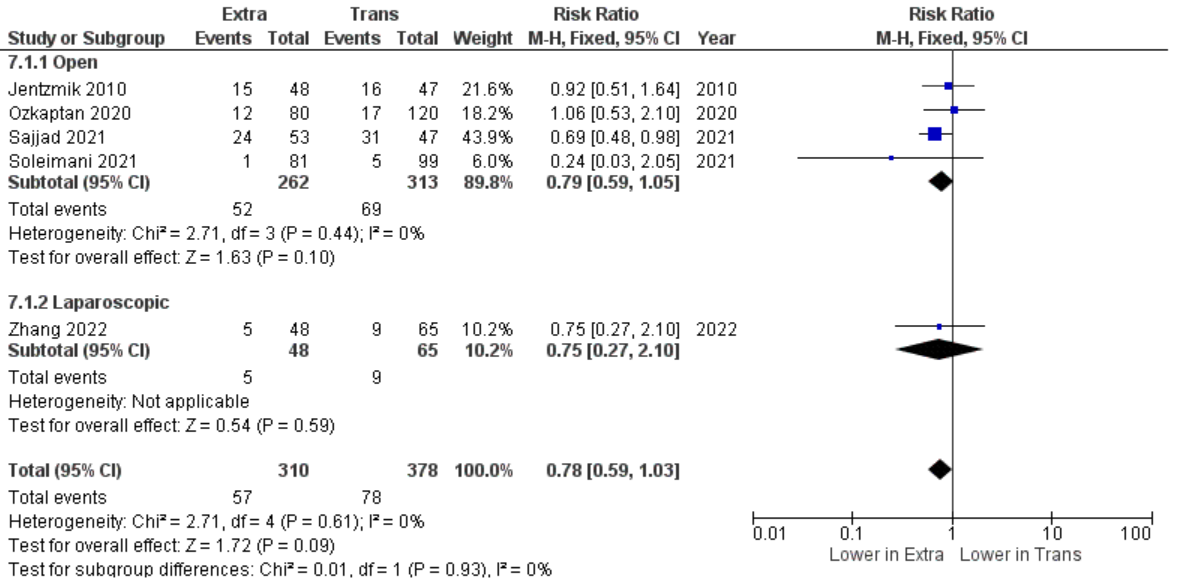
**

**Fig 6.** Major complications (n) in extraperitoneal vs transperitoneal approach. CI, confidence interval; IV, inverse variance; SD, standard deviation
