# Supplementary material for: Transperitoneal vs extraperitoneal radical cystectomy: A systematic review and meta-analysis
Source: PLoS One. 2023 Nov 30;18(11):e0294809. doi: 10.1371/journal.pone.0294809 (PMC10688672; doi:10.1371/journal.pone.0294809)
Supplement: S3 Fig — (DOCX) [file pone.0294809.s003.docx]

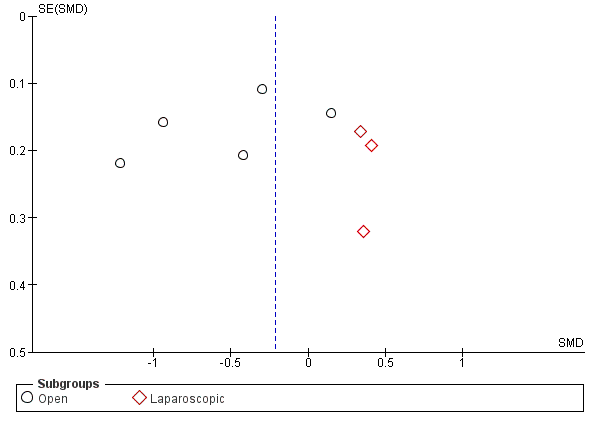


**Figure 1. Funnel Plot Operative Time**

**
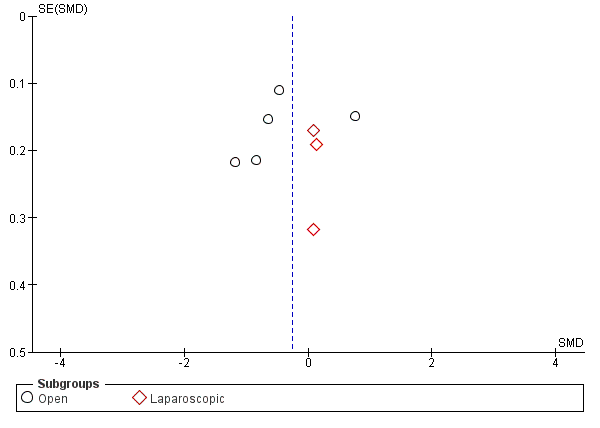
**

**Figure 2. Funnel Plot Estimated Blood Loss**

**
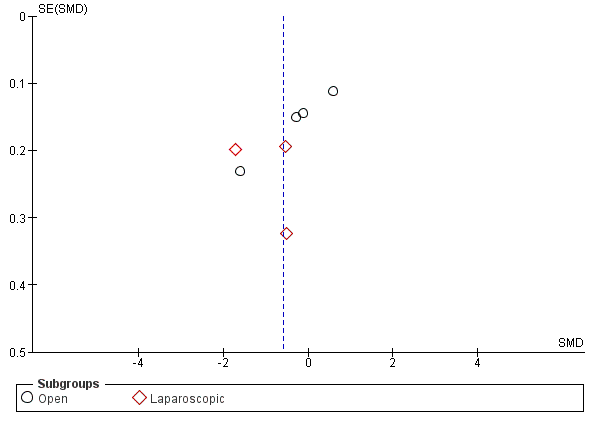
**

**Figure 3. Funnel Plot Hospital Stay**


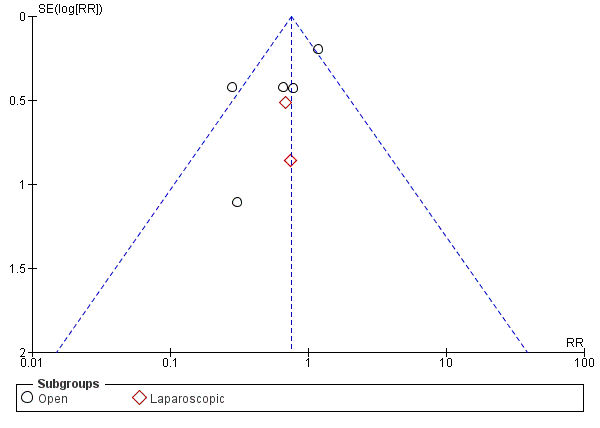


**Figure 4. Funnel Plot Total Infection**


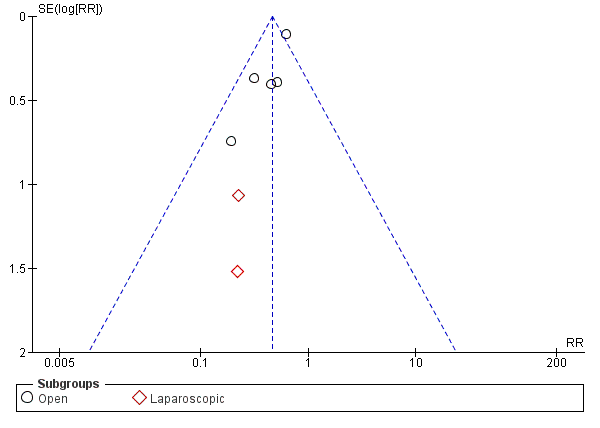
**Figure 5. Funnel Plot Post Operative Ileus**


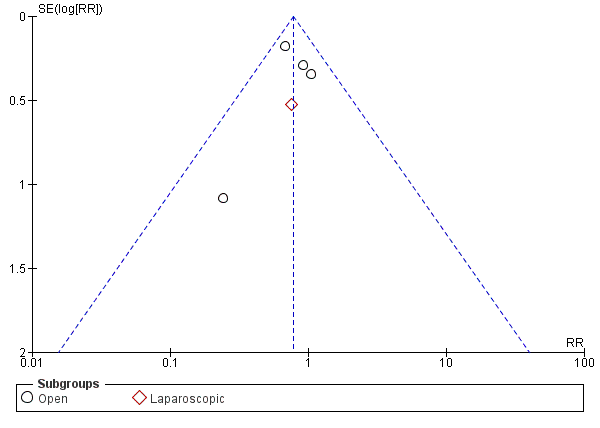


**Figure 6. Funnel Plot Major Complication**
