# Supplementary material for: Transperitoneal vs extraperitoneal radical cystectomy: A systematic review and meta-analysis
Source: PLoS One. 2023 Nov 30;18(11):e0294809. doi: 10.1371/journal.pone.0294809 (PMC10688672; doi:10.1371/journal.pone.0294809)
Supplement: S1 Table — (DOCX) [file pone.0294809.s004.docx]

**S1 Table.** **Literature search strategy**

| Database | Keywords | Result | Date and Time of Attempt |
| --- | --- | --- | --- |
| PubMed | Radical Cystectomy AND (Retrograde OR Extraperitoneal OR EPRC) AND (Transperitoneal OR Antegrade OR TPRC OR Intraperitoneal) AND Comparison | 34 | until August 31^st^ 2022 |
| Cochrane Library | “Transperitoneal” OR “Antegrade” OR “TPRC” OR “Intraperitoneal” OR “Retrograde” OR “Extraperitoneal” OR “EPRC” AND “Comparison” AND “Radical Cystectomy” | 72 | until August 31^st^ 2022 |
| Science Direct | Radical Cystectomy AND (Extraperitoneal OR EPRC) AND (Transperitoneal OR TPRC) AND Comparison | 144 | until August 31^st^ 2022 |
