# Supplementary material for: Transperitoneal vs extraperitoneal radical cystectomy: A systematic review and meta-analysis
Source: PLoS One. 2023 Nov 30;18(11):e0294809. doi: 10.1371/journal.pone.0294809 (PMC10688672; doi:10.1371/journal.pone.0294809)
Supplement: S2 Table — (DOCX) [file pone.0294809.s005.docx]

**S3 Table. Study Characteristics and Results**

| **Author** | **Year** | **Study**  **Design** | **Method** | **Sample Population** | **Number of Samples** | | **Age** | | **Parameter** | **Group** | |
| --- | --- | --- | --- | --- | --- | --- | --- | --- | --- | --- | --- |
|  |  |  |  |  | **E** | **T** | **E** | **T** |  | **E** | **T** |
| Feng, et al.^18^ | 2016 | Retrospective | Laparo | BC patients who underwent ELRC or TLRC with ureterocutaneostomy (Median follow up 13.8 ± 8 months and up 18.2 ± 10 months for Extraperitoneal and transperitoneal group) | 19 | 21 | 78.4 | 79 | Operative Time (Min) | 179.9 ± 38.3 | 165.6 ± 40.0 |
|  |  |  |  |  |  |  |  |  | EBL (mL) | 280.0 ± 111.1 | 271.9 ± 105 |
|  |  |  |  |  |  |  |  |  | Hospital Stay (Days) | 8.2 ± 1.6 | 9.5 ± 3.1 |
|  |  |  |  |  |  |  |  |  | Total Infection (n) | 2 | 3 |
|  |  |  |  |  |  |  |  |  | Pyelonephritis | 1 | 1 |
|  |  |  |  |  |  |  |  |  | Pneumonia | 1 | 2 |
|  |  |  |  |  |  |  |  |  | Post-Op Ileus (n) | 0 | 2 |
|  |  |  |  |  |  |  |  |  | Lymphorrhagia (n) | 1 | 1 |
|  |  |  |  |  |  |  |  |  | Major Comp (CD Grade 3-5) (n) | 0 | 0 |
| Jentzmik, et al.^13^ | 2010 | Retrospective | Open | Invasive urothelial cancer patients who underwent radical cystectomy with ileal neobladder  (Mean follow up= 37 months) | 48 | 47 | 64 | 66 | Staging |  |  |
|  |  |  |  |  |  |  |  |  | ≤T2 | 22 | 25 |
|  |  |  |  |  |  |  |  |  | ≥T3 | 26 | 22 |
|  |  |  |  |  |  |  |  |  | Operative Time (Min) | 377.4 | 405.4 |
|  |  |  |  |  |  |  |  |  | EBL (mL) | 363 | 412 |
|  |  |  |  |  |  |  |  |  | Post-Op Ileus (n) | 2 | 10 |
|  |  |  |  |  |  |  |  |  | Total Infection (n) | 8 | 10 |
|  |  |  |  |  |  |  |  |  | Pyelonephritis | 1 | 1 |
|  |  |  |  |  |  |  |  |  | Sepsis | 3 | 5 |
|  |  |  |  |  |  |  |  |  | Intraabdominal/pelvic abscess | 1 | 0 |
|  |  |  |  |  |  |  |  |  | Pneumonia | 3 | 4 |
|  |  |  |  |  |  |  |  |  | Wound dehiscence (n) | 7 | 6 |
|  |  |  |  |  |  |  |  |  | DVT (n) | 1 | 1 |
|  |  |  |  |  |  |  |  |  | PE (n) | 0 | 1 |
|  |  |  |  |  |  |  |  |  | Major Comp (CD Grade 3-5) (n) | 15 | 16 |
|  |  |  |  |  |  |  |  |  | Charlson score ≥3 | 29 | 27 |
| Kulkarni, et al.^8^ | 2018 | Retrospective | Open | BC patients who underwent radical cystectomy with ileal conduit & neobladder  (Mean follow up = 70 months) | 180 | 158 | 63 | 61 | Staging |  |  |
|  |  |  |  |  |  |  |  |  | T1,T2 | 83 | 84 |
|  |  |  |  |  |  |  |  |  | T3 | 97 | 74 |
|  |  |  |  |  |  |  |  |  | Operative Time (Min) | 284.94 | 310.6 |
|  |  |  |  |  |  |  |  |  | EBL (mL) | 343 | 375 |
|  |  |  |  |  |  |  |  |  | Hospital Stay (Days) | 7 | 6 |
|  |  |  |  |  |  |  |  |  | Post-Op Ileus (n) | 9 | 25 |
|  |  |  |  |  |  |  |  |  | Wound major dehiscence/ Healing disorder (n) | 5 | 5 |
|  |  |  |  |  |  |  |  |  | Bowel Leak (n) | 2 | 4 |
|  |  |  |  |  |  |  |  |  | Urine Leak (n) | 13 | 18 |
|  |  |  |  |  |  |  |  |  | Total Infection (n) | 9 | 12 |
|  |  |  |  |  |  |  |  |  | Pyelonephritis | 2 | 2 |
|  |  |  |  |  |  |  |  |  | Pneumonia | 7 | 10 |
|  |  |  |  |  |  |  |  |  | DVT (n) | 2 | 2 |
|  |  |  |  |  |  |  |  |  | PE (n) | 1 | 1 |
|  |  |  |  |  |  |  |  |  | Charlson score | 4 | 4 |
| Özkaptan, et al^5^ | 2020 | Retrospective | Open | BC patients who underwent RC and ileal conduit. Excluding orthotopic neobladder patients.  (Mean follow up = 70 months) | 80 | 120 | 65 | 65 | Staging |  |  |
|  |  |  |  |  |  |  |  |  | Ta, Tcis | 7 | 7 |
|  |  |  |  |  |  |  |  |  | T1 | 7 | 7 |
|  |  |  |  |  |  |  |  |  | T2 | 17 | 34 |
|  |  |  |  |  |  |  |  |  | T3 | 28 | 49 |
|  |  |  |  |  |  |  |  |  | T4 | 21 | 23 |
|  |  |  |  |  |  |  |  |  | Operative Time (Min) | 240 | 230 |
|  |  |  |  |  |  |  |  |  | EBL (mL) | 700 | 650 |
|  |  |  |  |  |  |  |  |  | Hospital Stay (Days) | 9 | 10 |
|  |  |  |  |  |  |  |  |  | Post-Op Ileus (n) | 7 | 23 |
|  |  |  |  |  |  |  |  |  | Total INFECTION (n) | 30 | 38 |
|  |  |  |  |  |  |  |  |  | Wound INFECTION | 13 | 16 |
|  |  |  |  |  |  |  |  |  | UTI | 8 | 12 |
|  |  |  |  |  |  |  |  |  | Fever | 4 | 4 |
|  |  |  |  |  |  |  |  |  | Urosepsis | 3 | 2 |
|  |  |  |  |  |  |  |  |  | Pyelonephritis | 1 | 1 |
|  |  |  |  |  |  |  |  |  | Gastroenteritis | 0 | 1 |
|  |  |  |  |  |  |  |  |  | Pneumonia | 1 | 2 |
|  |  |  |  |  |  |  |  |  | DVT (n) | 2 | 3 |
|  |  |  |  |  |  |  |  |  | PE (n) | 2 | 3 |
|  |  |  |  |  |  |  |  |  | Major Comp (CD Grade 3-5) (n) | 12 | 17 |
|  |  |  |  |  |  |  |  |  | Charlson score | 4 | 3 |
| Soleimani, et al^12^ | 2021 | Retrospective | Open | BC patients who underwent RC with ileal orthotopic and conduit diversions  (Mean follow up = 1 month) | 81 | 99 | 67.5 | 70 | Staging |  |  |
|  |  |  |  |  |  |  |  |  | T0N0 | 2 | 1 |
|  |  |  |  |  |  |  |  |  | T1N0 | 18 | 24 |
|  |  |  |  |  |  |  |  |  | T1N1 | 7 | 0 |
|  |  |  |  |  |  |  |  |  | T2N0 | 16 | 26 |
|  |  |  |  |  |  |  |  |  | T2N1 | 8 | 11 |
|  |  |  |  |  |  |  |  |  | T3aN0 | 1 | 0 |
|  |  |  |  |  |  |  |  |  | T3aN1 | 1 | 6 |
|  |  |  |  |  |  |  |  |  | T3Bn1 | 0 | 2 |
|  |  |  |  |  |  |  |  |  | T3aN2 | 1 | 0 |
|  |  |  |  |  |  |  |  |  | T4aN0 | 0 | 1 |
|  |  |  |  |  |  |  |  |  | T4aN1 | 0 | 0 |
|  |  |  |  |  |  |  |  |  | Operative Time (Min) | 262.8±37.2 | 298.2 ± 37.8 |
|  |  |  |  |  |  |  |  |  | EBL (mL) | 1310 | 1630 |
|  |  |  |  |  |  |  |  |  | Hospital Stay (Days) | 8.72 ± 4.02 | 10.11 ± 5.78 |
|  |  |  |  |  |  |  |  |  | Post-Op Ileus (n) | 8 | 19 |
|  |  |  |  |  |  |  |  |  | Wound Infection | 6 | 19 |
|  |  |  |  |  |  |  |  |  | Urine Leak | 7 | 14 |
|  |  |  |  |  |  |  |  |  | Major Comp (CD Grade 3-5) | 1 | 5 |
| Zhang, et al^3^ | 2022 | Retrospective | Laparo | BC (stage T1-T3) patients who underwent laparoscopic RC with intracorporeal urinary diversion  (Mean follow up = 15 months) | 48 | 65 | 67.8 | 66 | Staging |  |  |
|  |  |  |  |  |  |  |  |  | Ta T1s | 5 | 9 |
|  |  |  |  |  |  |  |  |  | T1 | 16 | 26 |
|  |  |  |  |  |  |  |  |  | T2 | 27 | 30 |
|  |  |  |  |  |  |  |  |  | TUR-BT | 14 | 38 |
|  |  |  |  |  |  |  |  |  | Operative Time (Min) | 286.5 ± 34.5 | 272.1 ± 35.5 |
|  |  |  |  |  |  |  |  |  | EBL (mL) | 405.2 ± 173.3 | 383 ± 180 |
|  |  |  |  |  |  |  |  |  | Hospital Stay (Days) | 12.7 ± 2 | 14 ± 2.7 |
|  |  |  |  |  |  |  |  |  | Post-Op Ileus (n) | 1 | 6 |
|  |  |  |  |  |  |  |  |  | Total Infection (n) | 5 | 10 |
|  |  |  |  |  |  |  |  |  | Pneumonia | 2 | 3 |
|  |  |  |  |  |  |  |  |  | Wound Infection | 2 | 4 |
|  |  |  |  |  |  |  |  |  | UTI | 1 | 2 |
|  |  |  |  |  |  |  |  |  | Pyelonephritis | 0 | 1 |
|  |  |  |  |  |  |  |  |  | Wound dehiscence/Healing disorder (n) | 3 | 2 |
|  |  |  |  |  |  |  |  |  | Major Comp (CD Grade 3-5) (n) | 5 | 9 |
|  |  |  |  |  |  |  |  |  | Urine Leak (n) | 1 | 1 |
|  |  |  |  |  |  |  |  |  | DVT (n) | 1 | 3 |
|  |  |  |  |  |  |  |  |  | PE (n) | 1 | 0 |
| Mirzagaleb, et al^19^ | 2021 | Retrospective | Laparo | Muscle-invasive bladder  cancer stage T2-3N0-2M0 who underwent RC  (Mean follow up = 70 months) | 79 | 62 | 63 | 61 | Operative Time (Min) | 142.1 | 127.5 |
|  |  |  |  |  |  |  |  |  | Hospital Stay (Days) | 7 | 12 |
|  |  |  |  |  |  |  |  |  | EBL (mL) | 375 | 320 |
|  |  |  |  |  |  |  |  |  | Total Infection (n) | 18 | 19 |
|  |  |  |  |  |  |  |  |  | Wound Infection | 6 | 9 |
|  |  |  |  |  |  |  |  |  | Sepsis | 1 | 0 |
|  |  |  |  |  |  |  |  |  | Pyelonephritis | 11 | 10 |
|  |  |  |  |  |  |  |  |  | Peritonitis(n) | 2 | 4 |
|  |  |  |  |  |  |  |  |  | Intra-Abdominal bleeding (n) | 2 | 3 |
|  |  |  |  |  |  |  |  |  | Stricture (n) | 1 | 0 |
|  |  |  |  |  |  |  |  |  | Adhesive intestinal obstruction (n) | 1 | 6 |
|  |  |  |  |  |  |  |  |  | Pulmonary artery thrombosis (n) | 4 | 3 |
|  |  |  |  |  |  |  |  |  | Charlson score | 4 | 4 |
| Sajjad, et al^14^ | 2021 | Retrospective | Open | BC patients who underwent RC with ileal conduits only. Excluding other diversion methods.  (Mean follow up = 1 month) | 53 | 47 | 61 | 58 | Staging |  |  |
|  |  |  |  |  |  |  |  |  | Non muscle invasive | 6 | 3 |
|  |  |  |  |  |  |  |  |  | Muscle invasive | 47 | 44 |
|  |  |  |  |  |  |  |  |  | Operative Time (Min) | 340.2±69.6 | 432±80.4 |
|  |  |  |  |  |  |  |  |  | EBL (mL) | 1130 ±480 | 1680 ±445 |
|  |  |  |  |  |  |  |  |  | Hospital Stay (Days) | 6.06±2.8 | 11.74±4.17 |
|  |  |  |  |  |  |  |  |  | Post-Op Ileus (n) | 33 | 47 |
|  |  |  |  |  |  |  |  |  | Total Infection (n) | 1 | 4 |
|  |  |  |  |  |  |  |  |  | Wound Infection | 1 | 3 |
|  |  |  |  |  |  |  |  |  | Intraabdominal abscess | 0 | 1 |
|  |  |  |  |  |  |  |  |  | Urine Leak (n) | 7 | 5 |
|  |  |  |  |  |  |  |  |  | Bowel Leak (n) | 0 | 1 |
|  |  |  |  |  |  |  |  |  | Major Comp (CD Grade 3-5) (n) | 24 | 31 |

Abbreviations: BC= Bladder Cancer, CD=Clavien Dindo, DVT=Deep Vein Thrombosis, E=Extraperitoneal, EBL=Estimated Blood Loss, ELRC=Extraperitoneal Laparoscopic Radical Cystectomy, EORC= Extraperitoneal Open Radical Cystectomy, LRC= Laparoscopic Radical Cystectomy, RC= Radical Cystectomy, T=Transperitoneal, TLRC= Transperitoneal Laparoscopic Radical Cystectomy, TUR-BT = transurethral resection of bladder tumor, UTI=Urinary Tract Infection.
