# Supplementary material for: Transperitoneal vs extraperitoneal radical cystectomy: A systematic review and meta-analysis
Source: PLoS One. 2023 Nov 30;18(11):e0294809. doi: 10.1371/journal.pone.0294809 (PMC10688672; doi:10.1371/journal.pone.0294809)
Supplement: S3 Table — (DOCX) [file pone.0294809.s006.docx]

**S5 Table. Risk of bias assessment (Newcastle–Ottawa Quality Assessment Scale criteria)**

| **Author** | **Representative-ness of the exposed cohort** | **Selection of the non-exposed cohort** | **Ascertainment of exposure** | **Demonstration that outcome of interest was not present at the start of the study** | **Comparability of cohorts based on the design or analysis** | **Assessment of outcome** | **Was follow-up long enough for outcomes to occur (≥30 days)** | **Adequacy of follow up of cohorts** | **Total Score**  **(Quality Score)** |
| --- | --- | --- | --- | --- | --- | --- | --- | --- | --- |
| Feng, et al. | - | ★ | ★ | ★ | ★★ | ★ | ★ | - | 7 (Good Quality) |
| Jentzmik, et al. | - | ★ | ★ | ★ | ★★ | ★ | ★ | ★ | 8 (Good Quality) |
| Kulkarni, et al. | ★ | ★ | ★ | ★ | ★★ | ★ | ★ | ★ | 9 (Good Quality) |
| Özkaptan, et al. | ★ | ★ | ★ | ★ | ★★ | ★ | ★ | - | 8 (Good Quality) |
| Soleimani, et al. | ★ | ★ | ★ | ★ | ★★ | ★ | ★ | ★ | 9 (Good Quality) |
| Zhang, et al. | ★ | ★ | ★ | ★ | ★★ | ★ | ★ | ★ | 9 (Good Quality) |
| Mirzagaleb, et al. | ★ | ★ | ★ | ★ | ★★ | ★ | ★ | - | 8 (Good Quality) |
| Sajjad, et al. | - | ★ | ★ | ★ | ★★ | ★ | ★ | ★ | 8 (Good Quality) |
| Zaytoun, et al. | ★ | ★ | - | ★ | ★ | - | ★ | - | 5 (Poor Quality) |
