# Supplementary material for: Transperitoneal vs extraperitoneal radical cystectomy: A systematic review and meta-analysis
Source: PLoS One. 2023 Nov 30;18(11):e0294809. doi: 10.1371/journal.pone.0294809 (PMC10688672; doi:10.1371/journal.pone.0294809)
Supplement: S4 Table — (DOCX) [file pone.0294809.s007.docx]

**S7 Table. PICO analysis**

| PICO | Analysis |
| --- | --- |
| P (Problem) | Radical Cystectomy |
| I (Intervention) | Extraperitoneal Approach |
| C (Comparison) | Transperitoneal Approach |
| O (Outcomes) | Operative time, Estimated Blood Loss, Hospital Stay, Post-Operative Ileus, Infection, Major Complication (Clavien-Dindo Grade 3-5) |
