# Supplementary material for: Transperitoneal vs extraperitoneal radical cystectomy: A systematic review and meta-analysis
Source: PLoS One. 2023 Nov 30;18(11):e0294809. doi: 10.1371/journal.pone.0294809 (PMC10688672; doi:10.1371/journal.pone.0294809)
Supplement: Attachment — Submitted filename: Response to reviewer.docx [file pone.0294809.s008.docx]

**Manuscript title : Transperitoneal vs extraperitoneal radical cystectomy: a systematic review and meta-analysis**

- : Author’s response/answer

**# Response to reviewer**

1. *It is very important to note that the reported complications on Radical cystectomy (RC) is significantly different in retrospective Vs. prospective studies. In honest prospective reports, the complication rates are between 60-80 percent of the patients. One of the paper included in your analysis-reference 9- has reported 0 patients with grade 3 and higher complications. In my view the authors and the publishing journals and the reviewers of this paper should be questioned and if the data is real, all of us should go there and get appropriate training from them. Until then, I do not think that this study should be included in your analysis. I am not sure about the quality of other papers included here as well.*
   - Thank you for you review for our manuscript. We have replaced the studies we used with only good quality retrospective studies. There are total 8 retrospective studies in this systematic review and meta analysis. We have already re-analyze and obtain the results listed in the revision of this manuscript (S3 Table. Study Characteristics and Results).
2. *Your studies probably matched the baseline criteria here, although I am not sure about it, but your paper does not show this. For example, are the groups have the same rate of Charlson comorbidity index, neoadjuvant chemotherapy rate, similar follow up period, baseline similar staging/variant rate, etc.*
   - We have some baseline characteristics to the table, such as staging and Charlson score (S3 Table. Study Characteristics and Results). Of the 8 studies we analyzed, the cancer stages in the studies used varied. The follow-up time of each study also differed from 1 to 70 months. Other characteristics were not described in the studies we analyzed.
3. *I do not think you need to review Clavien-Dindo Classification in your discussion. This is well known criteria and you can just cite the reference.*
   - Thank you for your feedback. We have removed the review Clavien-Dindo Classification in our discussion.
4. *I think hospital stay and risk of readmission is probably a better surrogate of all outcome measures here due to heterogeneity. You did not mention the readmission rate.*
   - We've tried to discuss this. However, from what we found from the studies we analyzed, there were no studies that explained the readmission rate.
5. *How many of patients in these studies followed ERAS protocol?*
   - Unfortunately, only 1 of 8 studies assessed follow-up of patients with the ERAS protocol (Zhang et al, 2022). ERAS protocol was not followed in this study which probably accounts for the varied duration of hospital stay.
6. *Your study regardless of my points has more limitation and it is better to be acknowledged by the authors rather than reviewers.*
   - Thank you for the feedback. We have add our limitation of this study because baseline staging and follow up time are quite varied. Therefor, bias in patient selection might be occurred.
