# Supplementary material for: Transperitoneal vs extraperitoneal radical cystectomy: A systematic review and meta-analysis
Source: PLoS One. 2023 Nov 30;18(11):e0294809. doi: 10.1371/journal.pone.0294809 (PMC10688672; doi:10.1371/journal.pone.0294809)
Supplement: Attachment — Submitted filename: RESPONSE TO REVIEWER - RESPONSE TO MINOR REVISION.docx [file pone.0294809.s009.docx]

Manuscript PONE-D-23-05792

- Author’s response / answer

*Reviewer #1: Thanks for addressing my points.*

*I like the way it is written now. I leave the following issues up to you if you want to address then in your discussion:*

*The data on readmission rate is lacking.*

*The ERAS protocol was used in one study, and the finding of that particular study is in line with the results of meta-analysis.*

We have responded to your comments below,

**# Response to reviewer 1**

Thank you for your constructive comments and suggestions for our manuscript, we are very pleased with your comments. We have revised the manuscript, we also have checked the comments, and revised the manuscript accordingly. We hope the revised version adds more value and is more suitable for publication.

Additional Editor Comments:

1. No introduction in the abstract

- Thank you for your comment. We have already changed the subtitle from Objectives to Background. The introduction of the manuscript is mentioned on that section

2. Submit PRISMA checklist as a supplement file

- Yes, we have added PRISMA checklist and PRISMA Abstract checklist as supplement file

3. Which meta-analysis model?

- The type of meta-analysis model that we used was The Cochran-Mantel-Haenszel Method

4. Why RR for report analysis? It is better used OR for cross sectional study

- Thank you for your comment. We chose Relative Risk due to the study design (cohort) that was included in this study.

5. Method of heterogeneity measurement and techniques of heterogeneity handling (sensitivity, subgroup analysis and meta regression)

- I^2^ was determined using RevMan version 5.4 in order to look into statistical heterogeneity

6. The research question (objectives) should be clearly addressed in accordance with PICO approach

- Thank you for your kind advice. We have mentioned our main objective is to compare the transperitoneal RC approach with the extraperitoneal RC approach in the treatment of bladder cancer patients based on some outcomes that can be seen as efficacy and safety profiles of both approaches

7. Publication biases?

- We have added funnel plots in the supplemental file as our way to check the publication biases

====================================================================

*1. Please ensure that you refer to Figure 1 in your text as, if accepted, production will need this reference to link the reader to the figure.*

*2. Please upload a copy of Figure 1. Or if the figure is no longer to be included as part of the submission please remove all references to it within the text.*

**# Response to Additional confirmation**

Thank you for your comments related to the missing Figure 1 issue. We are really sorry to inform you that Figure 1 has been changed to S2_Figure in the Supplemental files folder (PRISMA Flow Diagram). So, it is no longer to be included as part of the manuscript. We would like to erase and remove all the references related to it within the text. Figure 2-7 in the text is modified to Figure 1-6 as the impact of the change itself. Thank you for your kind understanding
